# Association of Oral Health and Cardiovascular Disease Risk Factors “Results from a Community Based Study on 5900 Adult Subjects”

**DOI:** 10.1155/2013/782126

**Published:** 2013-07-09

**Authors:** Hamid Najafipour, Tayebeh Malek Mohammadi, Foad Rahim, Ali Akbar Haghdoost, Mitra Shadkam, Mahdi Afshari

**Affiliations:** ^1^Physiology Research Center, Kerman University of Medical Sciences, Kerman, Iran; ^2^Oral and Dental Diseases Research Center, Kerman University of Medical Sciences, Kerman, Iran; ^3^Dental Public Health Department, School of Dentistry, Shafa Street, Kerman 76175, Iran; ^4^Research Center for Modeling in Health, Institute for Futures Studies in Health, Kerman University of Medical Sciences, Kerman, Iran

## Abstract

*Objectives*. This study aimed to determine the association between some oral health status as a risk factor for cardiac diseases and other cardiovascular disease (CVD) risk factors in a sample of Iranian population in 2011. *Methods*. The study recruited 5900 inhabitants who aged 15–75 years old of Kerman city through a population based cluster sampling. Having collected informed consent, participants were interviewed for CVD risk factors. Some oral health indicators such as DMFT, Gingival Inflammation index, and Community Periodontal Index were assessed. The association between oral health indices and CVD risk factors was tested using multivariate regression models. *Results.* The mean age of participants was 33.5 years, and 45.1% were male. Moderate gingival inflammation was observed in 67.6% of participants. Presence of sub- or supragingival calculus was more common (90%) in participants. Older age (RR from 2.7 to 3.88), cigarette smoking (RR = 1.49), and high blood glucose (RR = 1.41) showed an increased risk for oral diseases after adjustment for different covariates including established CVD risk factors. *Conclusion*. The study results showed an increase in periodontal diseases in the presence of some CVD risk factors. Therefore there may be a bilateral but independent association for both conditions and common risk factor approach preventive program is highly recommended.

## 1. Introduction

It is estimated that three-quarter of the global burden of cardiovascular disease (CVD) occurs in developing countries [1]. Sadly, it seems that in most of developing countries the mortality and morbidity due to CVD would have a sharp increase in the following decades because of the high prevalence of risk factors and also aging of their populations.

Iran, a developing country with more than 70 million population, is also facing with the epidemic of CVD [2]. The prevalence of coronary artery disease is as high as 20% in its capital, Tehran [3]. Myocardial infarction is among the first top three causes of death [4], and based on the results of a dynamic model, it seems that around 53 myocardial infarction per million population occurs daily in Iran [5].

CVD has a complex etiology determined by numerous risk factors that are strongly affected by genetic, socio-economic, and environmental variables [6]. In between, the role of inflammation in CVD has been investigated in many contexts in recent years and increasing body of evidence is being gathered in favor of the role of inflammation in the pathogenesis of CVD [7]. In this regards mouth pathogens and periodontal disease have been proposed as CVD risk factor [8].

Oral health is an integral part of general health and periodontal disease is a common chronic infection associated with a moderate systemic inflammatory response [9]. A national survey of periodontal health status of 3100 Iranian adult population aged 35–44 in 2002 showed that more than 55% had periodontal diseases [10]. Some epidemiological studies examined the association between oral health and CVD and they reported that people with periodontal disease are at increased risk up to 25% for CVD [11]. A research indicated that 84.4% individuals with CVD had periodontal disease compared to only 22.5% in individuals without CVD [12]. A recent meta-analysis concluded that periodontal disease and poor oral health could act as a risk factor to the pathogenesis of CVD [13]. However the relevance of oral health and CVD is not yet established, but association between dental hygiene and CVD risk factors has been reported by Frisbee et al. This study was based on self-reported oral hygiene behavior [14] which did not consider clinical oral examination to investigate the association of different risk factors for both issues.

Therefore this study aimed to examine the association of oral health status (gingival inflammation, periodontal health, presence or absence of microbial plaque, and dentition status) with CVD risk factors in a relatively large and wide-range age of Iranian population.

## 2. Study Population and Methods

This report is part of a study on CVD risk factors in Kerman (KERCADR Study), the largest province in South-east of Iran. The study recruited 5900 people aged 15–75 years old through one stage cluster sampling. Details of sampling and sample size calculation have been described in the first preliminary report of the study [15]. Concisely, 250 postal codes (as clusters) were selected randomly among an updated roster of four residential areas in provincial post office. The research coordinator team attended households in clusters. After briefing the household's member, all the eligible members (15–75 years old) were listed in the Kish Household Coversheet and recruited to the study. In the case of any household being absent for twice, another neighborhood household from the right was recruited. The recruitment was continued to reach 24 subjects in each cluster.

All recruited people were given an appointment card having the date, time, and the place of attending collaborating clinic for blood sampling, face to face interview, and clinical and dental examination. They were asked to fast for 12–14 hours before the appointment time in the morning and bring their medicines with themselves. Demographic data were collected (a minimum of one-year residency in Kerman was as an inclusion criteria for entering the study). Data regarding CVD risk factors such as behavioral risk factors, smoking and addiction status, and biological risk factors, hypertension and diabetes, depression and anxiety were collected through clinical examination and a piloted multipage questionnaire using some standard validated charts. Most of the mentioned measurements details are accessible in the first preliminary report of the study [15]. In brief the measurements included, Severe Anxiety (Beck anxiety questionnaire: BAI score > 25), severe depression (Beck depression questionnaire: BDI Score > 46), opium addiction (based on DSM4 criteria), daily cigarette smokers (more than 5 cigarettes per day), hypertension (BP ≥ 140/90 mmHg or taking drugs), overweight/obesity (BMI 25–29.9/30 or higher), diabetes (FBS ≥ 126 mg/dL or taking drug or Insulin), cholesterolemia (Chol > 200 mg/dL), and triglyceridemia (TG > 200 mg/dL). 

Oral examination was performed by a trained dentist under light using a dental mirror and WHO (CPI) probe. Presence of microbial plaque, presence of natural teeth or using dentures, any sign of infected teeth, and number of teeth with infection were recorded. WHO' criteria and codes were used for assessing dentition status [16]. Decayed, missing due to caries, and filled teeth (DMFT) index for each person was calculated. Gingival health status was assessed by standard Silness and Leo gingival inflammation index (GI) [17]. GI was scored as follows: mild (score = 0.1–1.0), moderate (score = 1.1–2.0), and severe (score = 2.1–3.0) inflammation. 

Periodontal health status was assessed by community periodontal index (CPI) and index' teeth were examined in the sextants [18]. CPI was scored as follows: 0 = healthy, 1 = bleeding on probing, 2 = supra- or subgingival calculus, 3 = pocket with 4-5 mm depth, and 4 = pocket > 6 mm depth. GI and CPI scores were calculated according to reference instruction [17, 18] and recorded on the relevant box on record sheet.

*Statistical Analysis*. Having adjusted the results for the clustering effect of sampling by using “survey commands” in Stata version 11, the level of oral health indices was explored in the whole samples and also in subgroups. Then, the levels of oral health indicators were modeled using multivariate poisson regression model; the exploratory variables, demographic variables, and known risk factors of CVD were entered using stepwise method.

## 3. Results

The mean ± SD age of participants was 33.5 ± 4 years from whom 45.1% were male. Visible plaque was observed in 99% of dentate survey' subjects and 33.3% of subjects had a chronic or acute dental infection with a mean number of 2.5 infected teeth. About 20% of subjects wear a full denture and 4.4% were edentulous with no denture. 

Moderate gingival inflammation (GI score = 2) and presence of calculus (CPI score = 2) were the most prevalent conditions among the participants. GI score was significantly different (*P* = 0.05) between men and women and also among different age groups (*P* < 0.0001). There was no difference between CPI scores of male and female groups, but it was significantly different (*P* < 0.001) among different age groups (Tables 1 and 2).

Mean DMFT of dentate subjects was 12.47 for all age groups and MT had the highest score (mean = 11.96) in 65–75 years age group, DT was higher (mean = 10.03) in 25–34 years age group, and 65–75 years age group had more filled (mean = 5) teeth (Table 3). There was a significant difference between DMFT of male and female participants (*P* = 0.003).

Table 4 shows the outcome of poisson regression model before and after adjustment of covariate including CVD risk factors. Risk of plaque accumulation was increasing with cigarette smoking (RR = 0.99) and opium addiction (RR = 0.99). Gingival inflammation showed an increase by factors such as increasing age (RR from 1.54 to 1.89). There was not a significant association between gingival inflammation and other CVD risk factors after adjustment for age, sex, and other risk factors.

Periodontal disease index (CPI) also showed an increase in age older than 35 years (RR from 2.7 to 3.88). Risk of periodontal disease increased just with raised fasting blood sugar (RR = 1.41) and cigarette smoking (RR = 1.49) when they were adjusted for multiple covariates including established CVD risk factors. 

Association of DMFT with other CVD risk factors was tested for participants less than 45 years old. There was a significant increase in the index in the presence of some risk factors such as opium addiction (RR = 2.06) and cigarette smoking (RR = 3.05).

## 4. Discussion

This study tries to explore the association between some oral health indicators as a risk factor for cardiac disease with some other CVD risk factors.

Results of the study showed that some background characteristics such as age could increase risk of oral diseases as they could increase risk of cardiac diseases. Findings of the study also revealed that some established CVD risk factors such as cigarette smoking, opium addiction, and high blood glucose increased risk of oral health problems even after adjustment for other factors. It has been shown that many factors such as age, tobacco use, and systemic conditions like diabetes are associated with the progression of periodontal disease and dental caries [19–22]. Results of the present study are in agreement with previous findings as mentioned factors showed more chance for increasing gingival inflammation and periodontal diseases when adjustment was applied for different risk factors.

A possible link between oral health and CVD was reported by some studies [23–25], but the link between oral hygiene and oral health is well established. Microbial plaque as an oral hygiene indicator has been identified as the main cause of periodontal and dental disease. Some studies linked oral disease to CVD through some reaction which could also happen with mouth diseases [7, 8]. These studies showed that microbial plaque, induced periodontal diseases, and dental infection could initiate an inflammatory response which could act as a risk factor for cardiac events. It is very important in this study as 99% of the study subjects had visible plaque on their teeth which could put them on a higher risk for inflammation and cardiac events. 

The findings of the present study are important from two points of view: firstly the data were collected from a general population sample as many studies have surveyed the association considering special risk groups or clinically diagnosed cardiac patients [26–28]. Secondly it seems that there are some common risk factors for oral diseases and CVD that contribute to severity of both conditions which have not been investigated.

So the study results for association of CVD risk factors with oral health condition as a risk factor for cardiac diseases could be interpreted independently. Because except cigarette smoking and diabetic condition as CVD risk factors which could affect oral health as it was confirmed by the study finding, other factors are not linked with oral health directly. However an indirect association and independent pathway is plausible.

It is understandable that most people with many background risk factors for cardiac diseases suffer from many problems which push their oral health and hygiene in the bottom of health priority table. As the results of current study showed many of those risk factors could affect oral health independently, in this regard a significant association between self-reported oral hygiene, systemic inflammation, and some CVD risk factors has been reported by Frisbee et al. [14]. However due to high sample size in the current study and budget restrictions we did not measure the markers of systemic inflammation and also screening of the population with cardiac diseases through clinical examination was not the goal of the study. But considering 70% of the subjects with gingival inflammation, periodontal disease and at least 99% with some degree of plaque accumulation on their teeth, how could leave them susceptible to inflammatory reactions and possible cardiac diseases.

Increasing risk for decayed or lost teeth in older age, with cigarette smoking and opium addiction is another important aspect of some common risk factors between oral diseases and CVD which was confirmed by the study results. 

A recent scientific statement from the American Heart Association has reviewed the possible link between oral health and CVD. The review showed that a casual relationship between two conditions could not be supported by observational studies. And also proposed that periodontal and cardiovascular diseases share multiple risk factors that are prevalent and powerful promoters of disease, including tobacco use, diabetes mellitus, and age. The results of present study confirmed this review' findings [29].

Therefore it can be concluded that many CVD risk factors were not associated to oral health status in this study but some common risk factors could be assumed for both conditions. These common factors could act independently as a risk factor for oral health and also for cardiac diseases as the study confirmed it. Both diseases are prevalent and receive major concern from public health aspect so preventive programs on the basis of common risk approach could be the best way to prevent the both issues.

## Figures and Tables

**Table 1:** Standardized prevalence of GI scores of males and females and in different age groups of participants.

GI score		1 (%)	2 (%)	3 (%)	*P *value
Sex	Male	27.4	69.7	2.9	**0.05**
	Female	31.7	65.4	2.9	
	Total	**29.6**	**67.6**	**2.8**	

Age groups	15–24	51.9	46.6	1.5	**<0.001**
	25–34	25.5	73.2	1.3	
	35–44	12.2	83.3	4.5	
	45–54	10.6	84.9	4.5	
	55–64	8.3	85.2	6.5	
	65–75	8.6	83.7	7.7	
	Total	**29.7**	**67.6**	**2.8**	

GI score: 1: mild inflammation, 2: moderate inflammation, and 3: severe inflammation.

**Table 2:** Standardized prevalence of CPI scores of males and females and in different age groups of participants.

CPI score		0 (%)	1 (%)	2 (%)	3 (%)	4 (%)	*P* value
Sex	Male	0.0	3.7	90.8	5.2	0.3	**0.34**
	Female	0.0	4.7	89.9	5.3	0.1	
	Total	**0.0**	**4.2**	**90.4**	**5.3**	**0.1**	

Age groups	15–24	0.0	6.5	90.7	2.7	0.1	**<0.001**
	25–34	0.0	4.9	91.6	3.4	0.1	
	35–44	0.0	1.7	90	8.3	0	
	45–54	0.0	1.8	90.5	7.3	0.4	
	55–64	0.0	1.2	86.1	12.3	0.4	
	65–75	0.0	2.1	86.3	11.4	0.2	
	Total	**0.0**	**4.2**	**90.4**	**5.3**	**0.1**	

CPI score: 0: healthy, 1: bleeding on probing, 2: supra- or subgingival calculus, 3: pocket 4-5 mm, and 4: pocket > 6 mm.

**Table 3:** Standardized mean of DMFT, DT, MT, and FT in males and females and in different age groups of participants.

Mean		DMFT	Decayed teeth (DT)	Missed teeth (MT)	Filled teeth (FT)
Sex	Male	12.18	8.89	4.55	3.17
	Female	12.76	9.37	4.70	3.8
	*P* value	**0.003**	**0.011**	**0.453**	**0.196**

Age group	15–24	9.48	8.89	2.12	2.76
	25–34	12.12	10.03	3.73	3.76
	35–44	14	9.31	5.53	3.62
	45–54	15.74	8.83	7.85	4.3
	55–64	17.24	7.98	9.95	4.18
	65–75	18.94	7.55	11.96	5
Total		**12.47**	**9.12**	**4.62**	**3.48**

**Table 4:** Association of oral health indicators with other CVD risk factors using multivariate adjusted regression.

CVD risk factors	Oral health indicators					
	Presence of plaque		GI scores (moderate and severe)		CPI scores (3 and 4)	
	Crude RR *P* value	Adjusted RR^*ð*^ (95% CL) *P* value	Crude RR *P* value	Adjusted RR^∞^ (95% CL) *P* value	Crude RR *P* value	Adjusted RR^§^ (95% CL) *P* value
Male*	1.0	1.0 (0.99–1.00)	0.96	0.93	1.06	1.05
Female	0.9	0.9	0.02	0.001	0.6	0.8

Age 15–24*						
25–34	0.99 0.08	0.99 (0.98–1.00) 0.09	1.54 <0.0001	1.54 (1.40–1.7) <0.0001	1.26 0.5	1.27 (0.51–3.15) 0.6
35–44	0.99 0.2	0.99 (0.98–1.00) 0.3	1.81 <0.0001	1.80 (1.64–1.97) <0.0001	3.10 <0.0001	2.7 (1.16–6.65) 0.02
45–54	0.98 0.001	0.98 (0.97–0.99) 0.00	1.85 <0.0001	1.83 (1.67–2.01) <0.0001	2.90 0.001	2.42 (1.00–5.85) 0.04
55–64	0.99 0.04	0.99 (0.98–1.00) 0.13	1.91 <0.0001	1.89 (1.73–2.07) <0.0001	4.81 <0.0001	3.88 (1.60–9.38) 0.003
65–74	0.99 0.2	0.99 (0.97–1.01) 0.5	1.92 <0.0001	1.87 (1.69–2.07) <0.0001	4.13 <0.0001	3.25 (1.25–8.40) 0.01

Cigarette smoking						
No*	0.99	0.99 (0.99–1.00)	1.13	1.03 (0.98–1.07)	1.52	1.49 (1.38–1.55)
Yes	0.1	0.02	<0.0001	0.17	0.02	0.05

Opium addiction						
Never* Dependent	0.99 <0.0001	0.99 (0.991–0.997) 0.00	1.18 <0.0001	1.01 (0.96–1.06) 0.4	1.53 0.06	1.04 (0.63–1.68) 0.9
On occasion	0.99 <0.0001	0.99 (0991–0.997) 0.00	1.10 0.002	0.97 (0.92–1.04) 0.5	1.31 0.3	1 (0.59–1.67) 0.9

Raised FBS	0.99	0.99	1.14	1.01 (0.97–1.05)	2.01	1.41 (1.01–1.95)
	0.3	0.2	<0.0001	0.4	<0.0001	0.04

Triglyceridemia (TG > 200)	0.99	0.998 (0.994–1.00)	1.07	0.97 (0.93–1.01)	1.31	0.99 (0.73–1.33)
	0.2	0.6	<0.0001	0.18	0.06	0.9

Cholesterolemia (Chl > 200)	0.99	0.998 (0.993–1.00)	1.13	1.01 (0.98–1.04)	0.95	0.68 (0.65–0.720)
	0.08	0.4	<0.0001	0.3	0.7	0.006

Overweight and obesity (BMI ≥ 30)	0.99	0.999 (0.994–1.00)	1.04	0.97 (0.94–1.01)	1.50	1.14 (0.85–1.50)
	0.3	0.9	0.01	0.1	0.003	0.4

Anxiety (yes)	1	1.00 (0.99–1.00)	1	1.02 (0.98–1.06)	N/A	N/A
	0.1	0.08	0.9	0.2		

Depression (yes)	0.99	0.997 (0.992–1.00)	0.96	0.99 (0.96–1.03)	N/A	N/A
	0.03	0.2	0.6	0.9		

*Reference group.

^*ð*^Adjustment for age, nutrition, education, and addiction status.

^*∞*^Adjustment for age, sex, smoking and addiction status, and nutrition.

^§^Adjustment for age, job, education, addiction, BMI, FBS, and lipid status.

N/A: not available.
